# Level and correlates of disrespect and abuse among newborns in selected public hospitals of Addis Ababa, Ethiopia

**DOI:** 10.1186/s12978-023-01673-1

**Published:** 2023-08-31

**Authors:** Rediet Gezahegn, Abiy Seifu Estifanos

**Affiliations:** Department of Reproductive, Family and Population Health, School of Public Health, Addis Ababa University, Addis Ababa, Ethiopia

**Keywords:** Disrespect and abuse, Respectful newborn care, Newborn care

## Abstract

**Background:**

The provision of respectful and dignified maternal and newborn care is an important component of the quality of childbirth care. Although a growing body of evidence was generated on disrespect and abuse (D&A) of women during childbirth in the past decade there is limited evidence on D&A experienced by newborns. Our study aimed to determine the level of and factors associated with D&A among newborns.

**Methods:**

We conducted the study in three public hospitals in Addis Ababa. We directly observed childbirth care starting from the first stage of labor through two hours after the birth of 498 mother–baby dyads. We used frequencies and percentages to describe different forms of D&A among newborns. We used binary and multivariable logistic regression analysis to assess the association between the D&A among newborns and independent variables.

**Result:**

All of the newborns 496/496 (100%) experienced at least one form of D&A. Physical abuse was experienced by 41.1% of newborns in the form of unnecessary airway suctioning (23.2%) or slapping or holding upside down (33.5%). Additionally, 42.3% weren’t dried immediately after birth, 9.1% weren’t placed on the mother’s abdomen skin-to-skin, 61.7% had their cord cut before 1 min of birth, 34.9% weren’t breastfed within an hour of birth, 24.2% didn’t receive vitamin K and 1.8% didn’t receive tetracycline. All newborns who developed complications (69/69) received treatments without the consent of parents/caregivers. Moreover, 93.6% of parents/caregivers didn’t receive explanations regarding newborn care while the lack of breastfeeding counseling and thermal support during the immediate post-partum period was 87.3%. The likelihood of D&A was higher among newborns who were preterm (AOR = 2.02; 95% CI: 1.11–3.69), female (AOR = 2.01; 95% CI: 1.37–2.95), delivered assisted by instrument (AOR = 2.19; 95%CI: 1.20–3.99), whose mothers reside in rural areas (AOR = 1.97; 95%CI: 1.22–3.20), born from unmarried mothers (AOR = 2.77; 95%CI (1.26–6.06) and whose mothers received fewer than four-time antenatal care (ANC) visits (AOR = 2.37; 95%CI: 1.42–3.96).

**Conclusion:**

Our study found a high magnitude D&A among newborns. Gestational age at birth, sex of the newborn, maternal residence, maternal marital status, number of ANC visits, and mode of delivery were statistically significantly associated with D&A among newborns.

## Abstract (plain language summary)

The provision of respectful and dignified care to mothers and newborns during antenatal, childbirth, and postnatal periods is crucial to improve their health outcomes and experiences. Although there is a growing body of knowledge on respectful maternity care, there is limited evidence that systematically assessed newborns’ experiences of care.

We measured the level of disrespect and abuse among newborns and assessed factors that increase the likelihood of experiencing disrespect and abuse among newborns born in three hospitals in Addis Ababa. We directly observed the care provision to assess whether the newborn was (1) physically abused (suction of the nose or mouth without medical indication; shaken, slapped, or held upside down), (2) stigmatized or discriminated (care compromised because the newborn was considered too sick to save, HIV exposed, or has congenital malformation), (3) didn’t receive standard care, and (4) mothers/caregivers didn’t receive counseling on breastfeeding and thermal care.

We found that all of the newborns have experienced at least one form of disrespect and abuse during childbirth or the postpartum period. 41% experienced physical abuse; 99.6% didn’t receive standard care; 87.3% didn’t receive counseling on breastfeeding and thermal care; and 13%, 75%, and 67% of the newborns who were identified as too sick to save, HIV exposed, and congenital malformation, respectively, were ignored, denied care or treated differently. In addition, the likelihood of experiencing disrespect and abuse was high among newborns who were preterm, female, from rural areas, of unmarried mothers, whose mothers received fewer than four ANC visits and delivered by instrument.

## Background

The World Health Organization (WHO) framework for the quality of maternal and newborn care consists of two interlinked dimensions; the quality of provision of care and the quality of experience of care by women, newborns, and their families. The framework also identified eight domains including the provision of evidence-based care, effective communication, respect and dignity, and emotional support [1]. The provision of respectful care for newborns is increasingly considered as an important component of providing dignified care. In 2019, the White Ribbon Alliance incorporated newborn babies in the updated respectful maternity care charter by recognizing that women and newborns have independent and inalienable basic human rights that must be respected and guaranteed [2].

Ethiopia is among the world’s top countries with the highest burden of neonatal mortality rate [3]. Between 2016 and 2019 the proportion of childbirths assisted by skilled providers in Ethiopia increased from 28 to 50%. However, during the same period neonatal mortality showed an increase from 29 to 33 per 1000 live births [4, 5]. This implies that unless the quality gap is closed through the provision of effective care for all women and newborns delivering in the facilities [6, 7] an increase in the proportion of births assisted by skilled attendants may not translate into saving lives [8]. Hence, the provision of high-quality care for women and newborns that addresses their rights, needs, experiences, and preferences is critical to attaining the national and Sustainable Development Goals (SDG) targets [7].

Women’s perception or experience of disrespect and abuse (D&A) during childbirth in the facility influences their choice about where to deliver and could prevent them from seeking health care services timely or at all increasing the risk of poor childbirth outcomes to themselves and their newborns [9–11]. Even though newborns can’t communicate verbally and give consent about their needs and preferences for tests, treatments, and referrals, their parents or caregivers have the right to receive information and provide informed consent for their child’s care in the child’s best interests. To address this the healthcare system should ensure the health, safety, and dignity of both the women and their newborns [2, 12].

D&A among newborns was defined only recently. In 2017, using the seven categories developed for the mistreatment of women during childbirth, a comprehensive systematic review was done to define D&A among newborns that identified eight categories. These are physical abuse, verbal abuse, stigma/discrimination, failure to meet professional standards of care, the poor rapport between patients and providers, health system conditions and constraints, legal accountability, and bereavement and posthumous care [13]. The systematic review offered a framework for assessing disrespect, abuse, and stigmatization among newborns.

Notwithstanding that, previous studies reported various forms of D&A among newborns including unnecessary suctioning of the airway [14, 15], slapping/holding the newborn upside down [15, 16], blaming of the mother for poor neonatal outcome [17], discrimination of small or human immunodeficiency virus (HIV) exposed newborns [18, 19], separation of the mother and newborn without medical indication [15, 20], provision of substandard essential newborn care (ENC) [21–23] and abandonment of health care [24]. Very few studies measured the coverage of respectful newborn care [25] and the level of mistreatment among newborns [14, 15, 20]. However, evidence that comprehensively assessed the level of D&A among newborns using direct observation of childbirth care is lacking. Our study aimed to determine the level of and factors associated with D&A among newborns through a comprehensive assessment of D&A among newborns using direct observation of childbirth care.

## Methods

### Study design and setting

We conducted a facility-based cross-sectional study in three public hospitals in Addis Ababa. The data were collected from 21 July to 18 September 2020. Based on United Nations estimates Addis Ababa had an estimated 4,794,000 population in 2020 [26]. The 2019 Mini Ethiopian Demographic and Health Survey (EDHS) report showed that 97% of the pregnant women in Addis Ababa received ANC at least once, 82% received four and more ANC visits, 96% were attended by a skilled provider during delivery, and 74% of mothers and newborns received a postnatal check within the first 2 days after birth [5].

For our study, we selected three hospitals located in Addis Ababa namely Gandhi Memorial Hospital (GMH), Tikur Anbessa Specialized Hospital (TASH), and Ras Desta Memorial Hospital (RDMH). GMH provides childbirth care services in nine separate rooms; each room has a single bed for labor follow-up and attending delivery. Whereas, TASH and RDMH provide childbirth care services in shared rooms with six and five beds, respectively, in the labor wards and four and three delivery couches, respectively, in the delivery wards. In the three hospitals, maternal and newborn health (MNH) care services are provided mainly by midwives, final-year medical students (interns), and obstetrics/gynecology resident physicians.

### Study participants

Study participants were mothers-baby dyads who delivered at the three hospitals during the data collection period. We included mothers who had live birth at the gestational age of 28 weeks and later and excluded mothers who had a stillbirth or live birth but whose neonates died within 2 h after delivery.

### Sample size determination

We used a single population proportion formula to calculate the sample size required to estimate the magnitude of D&A among newborns. Since there is no previous estimate, we assumed that 50% of newborns experience at least one form of D&A, 95% confidence interval (CI), and 5% margin of error. By considering a 10% non-response rate, we estimated a sample size of 427. To measure the association of exposure variables with D&A among newborns, we used a double population formula using variables previously identified to have an association with D&A among mothers. We considered the exposure variable (monthly income) that gave us the largest sample size (n = 544) using assumptions including 95% CI, 80% power, 10% non-response rate, and an adjusted odds of 1.74 (the odds of experiencing D&A among mothers with a monthly income of < 2000 Ethiopian Birr (ETB) was 1.74 times compared to mothers who earn 2000 or more ETB) [27]. We took 544 as our final sample size.

### Sampling procedure and data collection

We selected the three hospitals based on the volume of deliveries they manage to capture variation in the level and experience of D&A. Accordingly, we selected GMH, TASH, and RDMH from public hospitals in the city based on the high, medium, and low volume of deliveries they manage, respectively. Based on the reports from Health Management and Information System (HMIS), GMH manages 900–1200 deliveries monthly, TASH 500–600, and RDMH 200–300. The allocation of the sample to the hospitals was made proportional to their delivery volume. Thus 314, 150, and 80 participants were allocated for GMH, TASH, and RDMH, respectively. Given the limited resources we had, we employed a convenience sampling technique to recruit mothers. In a situation where more than one mother who fulfills the inclusion criteria were admitted to the labor ward, we selected one of the mothers randomly using a lottery method. When the observation for the selected mother was completed, we repeated the same process for the next mother.

We used a standard observation tool to directly observe forms of D&A among newborns. Based on Emma Sacks’s systematic review definition of D&A among newborns; the observation tool included four categories (physical abuse, stigma/discrimination, failure to meet professional standard of care, and poor rapport between patients and providers). Each of the categories has verification items [13]. We excluded other domains for logistics reasons. To capture the provision of recommended evidence-based newborn health care services more broadly, we added to the observation tool additional items including drying immediately after birth, cord care, administration of vitamin K and tetracycline eye ointment, initiation of skin-to-skin contact and breastfeeding within an hour after birth from the WHO safe childbirth checklist [28] and ENC guidelines [29]. We used a structured questionnaire that was initially prepared in English and translated into Amharic to interview mothers to collect information about the socio-demographic and obstetric characteristics of mothers. We also extracted the health-related characteristics of newborns and obstetric characteristics of mothers from their medical records.

Six data collectors, four male and two female nurses with Bachelor of Science (BSc) degree training, who were not staff of the selected public hospitals collected the data through direct observation of care, interview of mothers, and extraction of data from the maternity registers and charts. Two data collectors were assigned to each of the three hospitals. RG supervised the data collection. The training was given to the data collectors for two days before data collection. The training covered the objective of the study, data collection ethics, and detailed contents of data collection tools and procedures. We conducted a pre-test of the tools and data collection procedure before the actual data collection. The observation was started during the first stage of labor and continued for two hours after birth. We informed the health facilities and MNH care providers about the objective of the study. However, to minimize observation bias, the data collectors didn’t show them the items included in the observation tools. Although the data collectors were health workers with experience in childbirth care, their role was intentionally limited to observing the care and collecting data; they did not engage in the provision of care or giving feedback to the health care providers or mothers.

### Data processing and analysis

The data were entered using EpiData V.4.0 software and exported to Stata V.14 software for cleaning and analysis. Frequencies, means, standard deviations, and percentages were calculated to describe socio-demographic and obstetric characteristics of mothers and health-related characteristics of newborns.

We coded the items for each domain of D&A as “0” if not practiced and “1” if practiced. To generate a composite variable for a specific domain of D&A; all of the items under the domain were added together. If a mother-newborn dyad experienced at least one of the items under the specific domain of D&A, that domain of D&A was considered as experienced.

For the multivariable logistic regression analysis; we performed factor analysis as an intermediate step to reduce the number of items used to assess D&A among newborns. We used a total of 19 items to assess D&A among newborns. In the initial factor analysis, we dropped six items. Three items were dropped because they had missed data for > 10% of the observations and the other three items were dropped because of lack of variance across observations. The remaining 13 items were loaded across five factors with an Eigenvalue of > 1. After Orthogonal rotation, Cronbach’s alpha test was performed for the 13 items to measure the internal consistency which yielded a reliability coefficient of 0.6038. For each of the retained factors, Cronbach’s alpha test was performed among items that had a factor loading of > 0.4 or <− 0.4. Except for Factor 1, which had a reliability coefficient of > 0.7, the other factors were dropped due to low-reliability coefficients. We used Factor 1 as a measure of our outcome variable for the multivariable logistic regression analysis. Five items loaded more on Factor 1 which we labeled as “Physical abuse and substandard care” or simply as D&A among newborns. We dichotomized the outcome variable by adding the score for the five items. Those who didn’t experience any of the items were categorized as ‘No D&A’ and those who did experience at least one of the items were categorized as ‘D&A’. Table 1 presents the interpreted factor, items that had a factor loading of > 0.4, and the value of factor loadings.

**Table 1:** Items of D&A among newborns which had a value of factor loading > 0.4

Items^a^	Items that loaded on Factor 1 “Physical abuse and substandard care”
Suctioning without medical indication	0.6169
Shaking or slapping or turning upside down the newborn	0.8093
The newborn is not dried immediately after birth and is not well covered with a clean and dry towel	0.5023
Vitamin K was not administered	0.8036
Crowded conditions, shared beds	0.7097

^a^Items which had factor loading > 0.4

We did binary and multivariable logistic regression analyses to assess the association between the independent variables and the outcome variable. The examined independent variables include; socio-demographic characteristics of the woman (age, marital status, residence, occupation, educational status), obstetric characteristics of the woman (parity, number of ANC visits, mode of delivery, length of labor, any complication), and health-related characteristics of newborns (sex, gestational age at birth, birth weight, any complication).

In the multivariable regression models, we included independent variables with p-value of < 0.25 in the binary logistic regression and variables which had a statistically significant association with D&A from the literatures. We checked for multicollinearity among the independent variables using the variance inflation factor. We included all independent variables in the models since all of them had a variance inflation factor of fewer than 5.

## Result

We invited a total of 544 mothers to participate in the study, and 498 consented to participate yielding a response rate of 498/544 (91.5%). During data cleaning, we discarded incomplete data from two participants. Thus, we analyzed the data from 496 mother-baby dyads.

### Socio-demographic characteristics of mothers

The mean age of the mothers was 27.2 years (SD = 4.5); 315 (63.5%) of mothers were 25–34 years old. The majority of mothers were married [440/496 (88.7%)], attended secondary school and above [289/496 (58.3%)], and were housewives [364/496 (73.4%)] (Table 2).

**Table 2:** Socio-demographic characteristics of mothers in selected public hospitals of Addis Ababa, Ethiopia, 2020 (n = 496)

Variables	Frequency	Percent
*Age*		
< 25	148	29.8
25–34	315	63.5
≥ 35	33	6.7
*Place of residence*		
Urban	380	76.6
Rural	116	23.4
*Religion*		
Orthodox	211	42.5
Muslim	186	37.5
Protestant	91	18.4
Catholic	2	0.4
Traditional	6	1.2
*Marital status*		
Unmarried	28	5.7
Married/living together	440	88.7
Divorced	22	4.4
Widowed	6	1.2
*Educational level*^a^		
No formal education	15	3.0
Primary school	192	38.7
Secondary school	219	44.2
Technical/vocational	44	8.9
Higher	26	5.2
*Occupation*		
Student	10	2.0
Housewife	364	73.4
Daily laborer	20	4.0
Private business	62	12.5
Employee	40	8.1
*Estimated household monthly income in USD (n = 411)*^b^		
< 56.7	109	26.5
≥ 56.7	302	73.5

^a^Educational level refers to the highest level of education attended, whether or not that level was completed

^b^85 missed values

### Obstetrics characteristics of mothers

Among the mothers we observed [312/496 (62.9%)] were first-time mothers. Almost all, [491/496 (99.0%)] had at least one ANC visit for their current pregnancy, [382/496 (77.8%)] had four or more ANC visits. The majority of mothers [413/496 (83.3%)] gave birth vaginally either spontaneously [279/496 (56.3%)] or assisted by forceps/vacuum [134/496, (27.0%). The remaining [83/496 (16.7%)] gave birth through Cesarean section. The mean stay at the hospital from admission until the end of the observation was 5.9 h (SD = 3.4) (Table 3).

**Table 3:** Obstetrics characteristics of mothers in selected public hospitals of Addis Ababa, Ethiopia, 2020 (n = 496)

Variables	Frequency	Percent
*Place of birth*		
Gandhi memorial hospital	284	57.3
Tikur Anbassa specialized hospital	142	28.6
Ras Desta memorial hospital	70	14.1
*Parity*^a^		
Nulliparous	312	62.9
Primiparous	113	22.8
Multiparous	58	11.7
Grand multiparous	13	2.6
*ANC visit*		
Yes	491	99.0
No	5	1.0
*No. of ANC visits (n = 491)*		
< 4	109	22.2
≥ 4	382	77.8
*Referral status*		
Self-referred	19	3.8
Referred from another facility	477	96.2
*Mode of delivery*		
Spontaneous vaginal delivery	279	56.3
Instrument-assisted vaginal delivery	134	27.0
Cesarean section	83	16.7
*Maternal complication*^b^		
Yes	75	15.1
No	421	84.9
*Length of labor at the facility*		
$\le$12 h	482	97.2
> 12 h	14	2.8
*HIV status*		
Positive	4	0.8
Negative	492	99.2

^a^Parity excludes the current childbirth

^b^Maternal complications that occurred during labor, childbirth, or within the first two hours after delivery were a genital tear, postpartum hemorrhage, antepartum hemorrhage, severe preeclampsia, eclampsia, and anemia

### Health-related characteristics of newborns

More than one-third [341/496 (68.8%)] of the newborns were born term (37–41 weeks) whereas [84/496 (16.9%)] and [71/496 (14.3%)] of them were born preterm (28–<37 weeks) and post-term (> 41 weeks), respectively. More than half, [259/496 (52.2%)] of the newborns were female, [94/496 (18.9%)] had low birth weight (≤ 2500gram) with a mean birth weight of 3,019gram (SD = 507). Sixty-nine [69/496 (13.9%)] newborns experienced complications within two hours after delivery (Table 4).

**Table 4:** Health-related characteristics of newborns in selected public hospitals of Addis Ababa, Ethiopia, 2020 (n = 496)

Variables	Frequency	Percent
*Gestational age*^a^		
28–36 weeks	84	16.9
37-41weeks	341	68.8
≥ 42 weeks	71	14.3
*Sex*		
Female	259	52.2
Male	237	47.8
*Birth weight in gram*		
< 2000	5	1.0
2000–2500	89	17.9
> 2500	402	81.1
*Congenital malformation*^b^		
Yes	3	0.6
No	493	99.4
*Complication*^c^		
Yes	69	13.9
No	427	86.1
*HIV status*^d^		
Exposed	4	0.8
Non-exposed	492	99.2

^a^Gestational age is presented in completed weeks

^b^Hydrocephalus, meningomyelocele, and meningocele

^c^Complication that occurred within the first two hours after delivery included asphyxia, respiratory distress syndrome, and meconium aspiration syndrome

^d^HIV status of the newborn represents; exposed and non-exposed if born from HIV positive and HIV negative mother respectively

### The magnitude of directly observed D&A among newborns

Our data showed that all of the newborns [496/496 (100%)] experienced at least one form of D&A. The most frequently observed form of D&A was non-consented treatment for newborns who developed complications [69/69 (100%)], no mother was requested for consent before the provision of treatment for her sick newborn baby. The next commonly observed forms of D&A were giving no information or explanation to parents or caregivers before or during the provision of ENC [464/496 (93.6%)], and lack of breastfeeding counseling and thermal support during the immediate postpartum period [433/496 (87.3%)].

Our data also showed that a large proportion of newborns were not given the recommended ENC: [210/496 (42.3%)] were not dried immediately, [306/496 (61.7%)] had their cord clamped within one minute after birth, [45/496 (9.1%)] were not placed on their mother’s abdomen skin-to-skin immediately after birth, and [173/496 (34.9%)] were not breastfed within one hour after birth. In addition, [120/496 (24.2%)] and [9 (1.8%)] of the newborns were not given Vitamin K and Tetracycline eye ointment, respectively. It is recommended that healthy mothers and newborns should receive care in the facility for at least 24 h, however, our study showed [296/496(59.7%)] of newborns discharged before 24 h. Moreover, [122/496(24.6%)] of newborns shared a bed and [51/496(10.3%)] of mothers of the newborns reported neglected or their care delayed.

Similarly, a significant proportion of the newborns [204/496(41.2%)] experienced at least one form of physical abuse in which 115/496 (23.2%) of newborns with clear amniotic fluid who started breathing on their own received routine airway suctioning and a third of newborns [166/496 (33.5%)] were slapped on their back or held upside down.

The magnitude of each item of D&A is shown in Table 5.

**Table 5:** Directly observed D&A among newborns in selected public hospitals of Addis Ababa, Ethiopia, 2020 (n = 496)

Categories of Disrespect and abuse	Yes	No
	n (%)	n (%)
*Physical abuse*	204 (41.1)	292 (58.9)
Airway suctioning without medical indication	115 (23.2)	381 (76.8)
Shaking or slapping or holding the newborn upside down	166 (33.5)	330 (66.5)
*Stigma and discrimination*		
Considering some ill babies “too sick to save”^a^	9 (13.0)	60 (87.0)
Discrimination against HIV exposed newborns^b^	3 (75.0)	1 (25.0)
Discrimination against newborns with congenital malformation^c^	2 (66.7)	1 (33.3)
*Failure to meet professional standards of care*	494 (99.6)	2 (0.4)
The newborn is not dried immediately after birth and is not well covered with a clean and dry towel	210 (42.3)	286 (57.7)
Newborn not placed on mother’s abdomen skin-to-skin	45 (9.1)	451 (90.1)
Cord cut within one minute	306 (61.7)	190 (38.3)
Vitamin K was not administered	120 (24.2)	376 (75.8)
Tetracycline eye ointment was not administered	9 (1.8)	487 (98.2)
Breastfeeding is not initiated within the first hour after birth	173 (34.9)	323 (65.1)
The newborn is separated from the mother/parent/caregiver without medical indication	0 (0.0)	496 (100.0)
Non-consented treatment of a newborn (consent from parents or caregiver)^a^	69 (100.0)	0 (0.0)
Lack of privacy or confidentiality especially related to the HIV status of infant private (talking about it in a way that others can hear)^b^	1 (25.0)	3 (75.0)
Crowded conditions, shared beds	122 (24.6)	374 (75.4)
Early discharge from the facility (before 24 h)	296 (59.7)	200 (40.3)
No explanation is given to parents/caregivers at least once about the healthcare services provided to newborn	464 (93.6)	32 (6.4)
Neglect or delay in the provision of care while the newborn needed care at any point during a hospital stay	51 (10.3)	445 (89.7)
*Poor rapport between patients and providers*	433 (87.3)	63 (12.7)
Parent/caregiver is not counseled on breastfeeding counseling and thermal support during the immediate postpartum period	433 (87.3)	63 (12.7)

^a^The denominator is newborns who experienced complication/s after delivery

^b^The denominator is newborns who were born from HIV-positive mothers

^c^The denominator is newborns who had congenital malformation/s

### Factors associated with the observed D&A of newborns

Of the total newborns observed, six in ten [298/496 (60.1%)] of them ‘received substandard care’ the outcome variable used to measure D&A among newborns.

The multivariable logistic regression analysis showed that the health-related variables of the newborn (gestational age at delivery and newborn’s sex), socio-demographic variables of the mother (place of residence and marital status), and obstetrics variables of the mother (number of ANC visits and mode of delivery) were found to have a statistically significant association with the outcome variable.

The odds of experiencing D&A among newborn was about two times more likely among preterm newborn (AOR = 2.02, 95%CI: (1.11–3.69)) compared to term newborns, among female newborns (AOR = 2.01, (1.37–2.95)) compared to male newborns, among newborns who were born from mothers who reside in rural areas (AOR = 1.97, 95%CI: 1.22–3.20) compared to those who reside in urban areas, among newborns delivered through instrument-assisted delivery (AOR = 2.19, (1.20–3.99) compared to those who were delivered through Cesarean section; 2.7 times more likely among newborns of currently unmarried mothers (AOR = 2.77, 95% CI:1.26–6.06) compared to those who were married; and 2.4 times more likely among newborns whose mothers received ANC visits fewer than four times (AOR = 2.37, 95% CI 1.42–3.96) compared to those who received four or more times (Table 6).

**Table 6:** Factors associated with observed D&A of newborns in selected public hospitals of Addis Ababa, 2020

Variables	Substandard care		COR 95% CI	AOR 95% CI
	No	Yes		
*Gestational age*				
28–36 weeks	18 (21.4)	66 (78.6)	2.77(1.58–4.88)	2.02 (1.11–3.69)*
37–41 weeks (Ref)	147 (43.1)	194 (56.9)	1	1
≥ 42 weeks	33 (46.5)	38 (53.5)	0.87(0.52–1.46)	0.92(0.54–1.58)
*Sex*				
Female	85 (32.8)	174 (67.2)	1.87(1.29–2.68)	2.01 (1.37–2.95)***
Male (Ref)	113 (47.7)	124 (52.3)	1	1
*Residence*				
Urban (Ref)	164 (43.2)	216 (56.8)	1	1
Rural	34 (29.3)	82 (70.7)	1.83(1.16–2.87)	1.97(1.22–3.20)**
*Marital status*				
Married (Ref)	189 (42.9)	251 (57.1)	1	1
Unmarried, divorced, widowed	9 (16.1)	47 (83.9)	3.93(1.88–8.22)	2.77(1.26–6.06)*
*No. of ANC*				
< 4	25 (22.9)	84 (77.1)	2.72(1.67–4.44)	2.37(1.42–3.96)**
≥ 4 (Ref)	171 (44.8)	211 (55.2)	1	1
*Mode of delivery*				
Spontaneous vaginal delivery	114 (40.9)	165 (59.1)	1.48(0.91–2.43)	1.37(0.81–2.31)
Instrument-assisted vaginal delivery	42 (31.3)	92 (68.7)	2.24(1.27–3.95)	2.19(1.20–3.99)**
Cesarean section (Ref)	42 (50.6)	41 (49.4)	1	1

Ref: Reference group

*p-value < 0.05

**p-value < 0.01

***<0.001

## Discussion

Our study assessed the level of and factors associated with D&A among newborns through direct observation of childbirth care in three hospitals in Addis Ababa. We found that all newborns experienced at least one form of D&A. The proportion of newborns who experienced specific forms of D&A ranged from 0 to 100%. Gestational age at birth, sex of the newborn, maternal residence, maternal marital status, number of ANC visits, and mode of delivery were significantly associated with experiencing D&A among newborns.

Previous studies reported a wide range of magnitude of D&A among newborns. In a study conducted in Nepal, none of the mothers reported their newborns experiencing any form of D&A during exit interviews [25]. However, there is a tendency to underreport negative experiences during exit interviews within the health facility compound. Studies conducted in Tanzania showed higher reports of D&A during household surveys and using direct observation compared with mothers’ reports documented through exit interviews [30, 31].

Our study used a checklist gleaned from the first comprehensive systematic review that defined D&A among newborns [13], WHO’s ENC guideline, and safe childbirth checklist [28, 29] and the updated respectful maternity care charter [2] to measure the various forms of D&A newborns might experience. The lack of consensus on what constitutes D&A among newborns and a standard measurement tool and data collection method makes the need to develop a standard validated measurement tool that comprehensively captures the different aspects of D&A among newborns more urgent.

The WHO recommends the provision of ENC immediately after birth including immediate drying, early skin-to-skin initiation, delayed cord care, early breastfeeding initiation, vitamin K and Tetracycline administration [29]. However, our study revealed a gap in the provision of these ENC. This is consistent with previous studies that reported a range of gaps in the provision of ENC components [14, 15, 21, 22]. Given Ethiopia’s ambitious plan to meet the SDG targets, our finding indicates the need for a concerted effort by all stakeholders to increase the coverage of quality ENC services to ensure all newborns benefit from the care.

In our study, potentially harmful newborn care practices such as routine airway suctioning and shaking or slapping, or holding the newborn upside down were observed among one-fourth and one-third of the newborns, respectively. Compared to our data a recent multi-country facility-based observational study reported a higher proportion of routine airway suctioning (68%) and a relatively lower proportion of slapping or upside-down holding of the neonates (19%) [15]. These practices were commonly practiced in previous times. However, currently, the WHO recommends against them because of inconclusive evidence regarding their benefits or their negative outcomes [32, 33]. Further studies exploring the reasons why care providers continue to practice the potentially harmful practices and designing effective interventions to reduce them are needed.

Even though newborns are not able to communicate, their parents or caregivers are entitled to receive information and provide informed consent for their child’s care in the child’s best interest [2]. Our study found that all of the mothers whose newborns needed treatment were not asked for consent [69/69 (100%)] before treatment was given to their newborns. This was high compared to studies from Kenya (60%) and Nepal (63%) [14, 20]. The very high rate of non-consented care observed in our study could be partly because it assessed whether consent has been sought before the provision of any type of care for newborns with complications while the study from Kenya assessed consent before procedures and examination and the Nepal study before medical interventions. In addition, our study showed that the majority of mothers [464/496 (94%)] didn’t receive any information or explanation about the routine care provided to their newborns. We argue that beyond the immediate unfavorable experience of mothers, these experiences might affect future healthcare-seeking and newborn care practices among mothers [20]. There is an urgent need for improving the communication between MNH providers and mothers or caretakers.

Our study also captured forms of D&A among newborns derived from health system deficiencies including crowded conditions/shared beds and the practice of discharging of newborns before 24 h after birth. These were linked to lack of space and a shortage of beds in the delivery rooms at the study hospitals. In addition, our data showed that one in ten newborns was neglected or received delayed care. This could be due to the high caseload at the study hospitals that overwhelm the MNH providers as a qualitative study finding from Kenya documented [34]. The health system should create an enabling environment for the health workers to provide quality care that ensures the safety and dignity of both the women and their newborns [2, 35].

In low-income countries, the smallest and sickest newborns particularly those born before 28 weeks of gestation, usually die [36] or are not at all considered viable. To improve their survival and well-being preterm newborns need extra care and attention during childbirth care and the postnatal period. Our data showed that preterm newborns were about two times more likely to experience D&A compared to term newborns. Given the pre-existing vulnerability of preterm newborns, failure to provide standard care would further reduce their chance of survival.

Our finding showed newborns whose mothers reside in rural areas and are currently unmarried were more likely to experience D&A compared to those living in urban areas and married. A significant association between a mother’s socio-demographic characteristics and the mistreatment of newborns was observed in a previous study in Nepal [14]. In addition to conducting further studies to explore reasons for mistreatment based on socio-demographic characteristics, interventions aimed at improving healthcare provider’s attitudes and acknowledging implicit biases need to be considered [37].

Our data also found that newborns whose mothers received fewer than four ANC visits were more likely to experience D&A compared to newborns whose mothers received four and more visits. Similarly, newborns delivered through instruments were more likely to experience D&A compared to those delivered through Caesarean section (C/S). Further studies exploring the reasons behind these are needed.

Our study has strengths and limitations. We believe the use of direct observation of childbirth and newborn care in our study provided us with a more objective assessment of D&A experiences among newborns. However, the direct observation might have also increased the likelihood of the healthcare providers behaving differently due to the observation. To minimize this, we did not show the specific checklist we used to assess the D&A and, it is also unlikely that they maintain their changed behavior during the relatively long observation period and throughout the data collection period. Although we intentionally included hospitals with different caseloads, our study was conducted at three facilities located in Addis Ababa limiting the representativeness of the data regarding newborn care provision at hospitals and health centers in the country. We also didn’t limit the number of observations per provider; one healthcare provider was observed more than once. Thus, we weren’t able to control the effect of health professional-related factors in our analysis. In addition, the convenience sampling technique that we employed limits the representativeness. For logistical reasons our observation duration lasted only until two hours after birth and, hence, our data did not capture the care provided afterward. Furthermore, due to the small sample size, we were not able to do a sub-analysis of the D&A experience for newborns with neonatal congenital malformation and who were HIV exposed. Finally, our observation didn’t categorize D&A assessment by the severity of complications some of the newborns experienced. This might inflate the D&A prevalence among these groups of the infant population as some of the recommended routine and/or additional care may be withheld or delayed to them due to urgent interventions they need. Thus, we recommend further research overcoming these limitations.

## Conclusion

Our study found a high magnitude D&A among newborns. The forms of D&A among newborns included the provision of substandard care and poor communication between mothers of newborns and the health care providers. Forms of D&A among newborns derived from health system deficiencies were also observed. Gestational age at birth, sex of the newborn, maternal residence, maternal marital status, number of ANC visits, and mode of delivery were significantly associated with the D&A. Interventions directed at the health system and individual level are recommended to improve the provision of respectful and dignified care to newborns.

## Data Availability

The datasets used and analyzed during the current study are available from the corresponding author upon reasonable request.

## Abbreviations

ANC: Antenatal care
AOR: Adjusted odds ratio
COR: Crude odds ratio
CI: Confidence interval
D&A: Disrespect and abuse
ENC: Essential newborn care
ETB: Ethiopian Birr
GMH: Gandhi Memorial Hospital
HIV: Human immunodeficiency virus
HMIS: Health Management and Information System
MNH: Maternal and newborn health
RDMH: Ras Desta memorial Hospital
SD: Standard deviation
SDG: Sustainable Development Goals
TASH: Tikur Anbessa Specialized Hospital
WHO: World Health Organization
